# SH003 activates autophagic cell death by activating ATF4 and inhibiting G9a under hypoxia in gastric cancer cells

**DOI:** 10.1038/s41419-020-02924-w

**Published:** 2020-09-02

**Authors:** Tae Woo Kim, Chunhoo Cheon, Seong-Gyu Ko

**Affiliations:** Department of Preventive Medicine, College of Korean Medicine, Kyung Hee University, Seoul, Korea

**Keywords:** Cancer therapeutic resistance, Drug development

## Abstract

In gastric cancer (GC), hypoxia is one of the greatest obstacles to cancer therapy. In this present study, we report that SH003, an herbal formulation, induces ER stress via PERK-ATF4-CHOP signaling in GC. SH003-mediated ER stress inhibits G9a, a histone methyltransferase, by reducing STAT3 phosphorylation and activates autophagy, indicating to the dissociation of Beclin-1 and autophagy initiation from Bcl-2/Beclin-1 complex. However, the inhibition of PERK and CHOP inhibited SH003-induced cell death and autophagy activation. Moreover, targeting autophagy using specific siRNAs of LC3B or p62 or the autophagy inhibitor 3-MA also inhibited SH003-induced cell death in GC. Interestingly, SH003 induces BNIP3-mediated autophagic cell death under hypoxia than normoxia in GC. These findings reveal that SH003-induced ER stress regulates BNIP3-induced autophagic cell death via inhibition of STAT3-G9a axis under hypoxia in GC. Therefore, SH003 may an important tumor therapeutic strategy under hypoxia-mediated chemo-resistance.

## Introduction

Gastric cancer is estimated to be one of the most common cancer types and is the third leading cause of cancers world-wide^1,2^. Cisplatin is a well-known drug for cancer therapy and is a candidate anti-cancer drug for therapy in various tumors^3^. However, because of the chemo-resistance and unexpected adverse effects, there are concerns with the use of cisplatin for therapy in cancer patients^4^. In addition, many anti-cancer drugs having toxic adverse effects can also kill normal cells, and thus, herbal medicine may be an effective strategy to reduce the side effects of anti-cancer drugs^5^. To overcome these problems, the use of natural compounds extracted from plants and animals may be a novel therapeutic strategy for cancer therapy^6^.

We previously reported a powerful herbal formula named SH003 with anti-cancer properties. It contained *Astragalus membranaceus* (Am), *Angelica gigas* (Ag), and *Trichosanthes Kirilowii Maximowicz* (Tk) in 1:1:1 ratio (w/w) in various cancers^7,8^. SH003 was reported as herbal medicine for benefits against cancer, such as anti-inflammation, anti-angiogenesis, and anti-tumor^9^. Triple-negative breast cancer (TNBC) cells were highly sensitive to SH003 through the induction of a p53-related protein called p73 protein and exerted synergic effect with doxorubicin, an anti-cancer drug^10,11^. SH003 activated autophagy by accumulating p62 via the inhibition of STAT3 and mTOR signaling in breast cancer and inhibited tumor growth and metastasis in vitro and in vivo^12^.

Autophagy, known as “self-eating”, is a quality control mechanism involving elimination of damaged proteins and organelles^13^. Recent studies suggest that autophagy plays dual roles in cell survival and death mechanism^14^. In tumor environment, autophagy has dual functions, including tumor suppression by autophagy deficiency and tumor promotion by limiting stress^15^. Autophagy induction during stimulation-induced apoptosis for cancer therapy can either be protective or be a cell death mechanism, and autophagy-mediated cell death could function by activating type-2 cell death^16^. Therefore, anti-cancer drug-caused excessive autophagy in tumor cells leads to autophagic cell death, and therapeutic strategy targeting autophagy revealed the usefulness of cancer therapy^17^.

Unfolded protein response (UPR) was induced by multiple stresses in tumor cells and by the activation of endoplasmic reticulum (ER) stress sensors implicated in the autophagy pathway^18^. The ER is highly sensitive to hypoxia stress, resulting in the accumulation of misfolded proteins in the ER lumen^19^. Prolonged hypoxia can induce autophagic cell death, and ER stress is required for autophagy activation^20^. The present study tried to identify the mechanism between ER stress and autophagic cell death by examining the changes in the PERK–ATF4–CHOP pathway and AMPK–ULK1–LC3B signaling in SH003-treated GC cells.

## Results

### SH003-induced cell death in GC cells

**Fig. 1:**
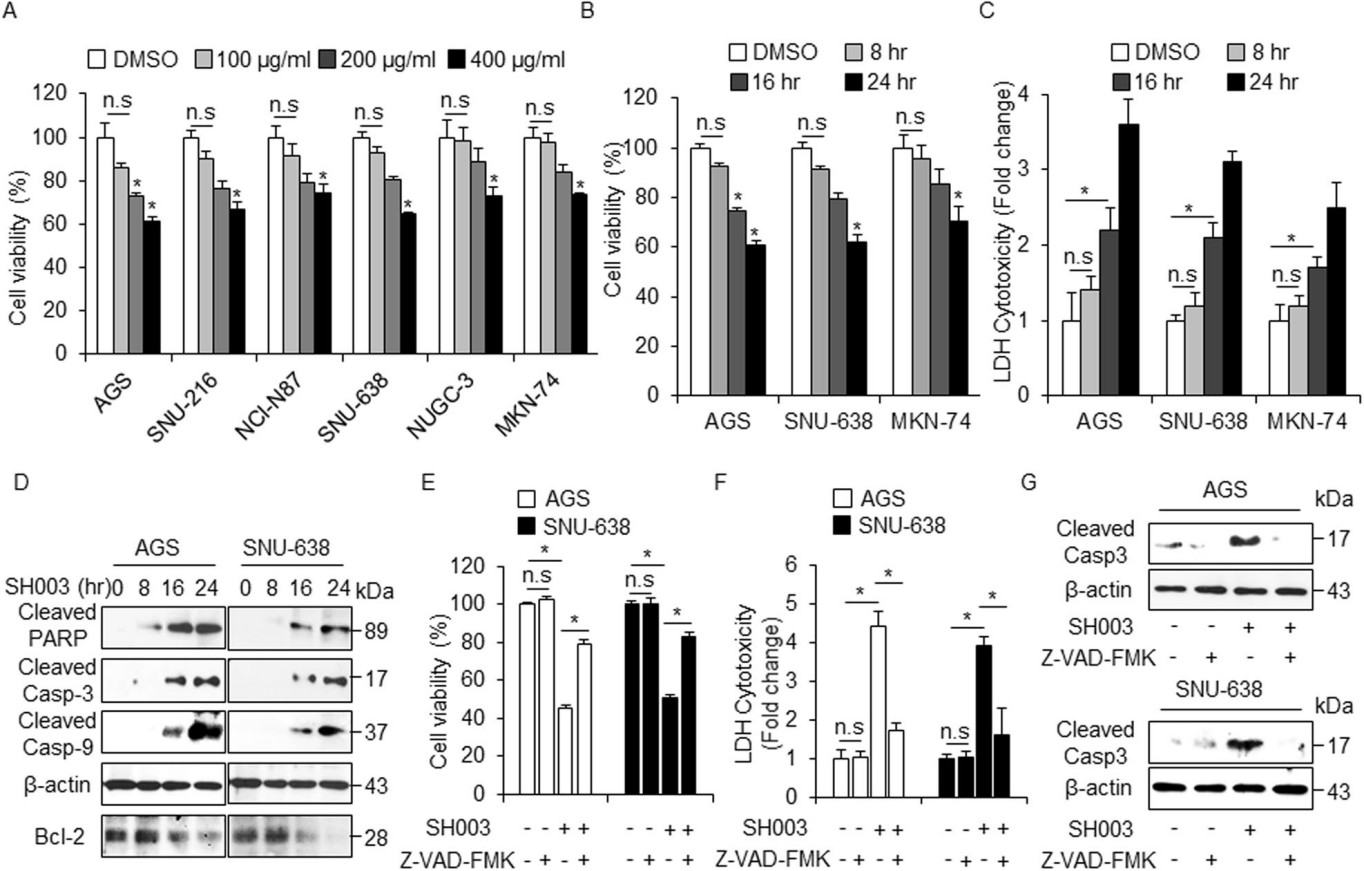
Cytotoxic effects of SH003 in GC cells. **a**, **b** Cell viability of SH003 in GC cells, including AGS, SNU-216, NCI-N87, SNU-638, NUGC-3, and MKN-74 were measured using WST-1 on 96-well plates, and SH003 was treated in a dose-dependent (0, 100, 200, and 400 µg/mL, 24 h) and time-dependent manner (0, 8, 16, and 24 h). Cell viability of the DMSO-treated cells was set at 100%; *p < 0.05. **c** The LDH activity from AGS, SNU-638, and MKN-74 cells with SH003 (400 µg/mL) in a time-dependent manner (8, 16, and 24 h); *p < 0.05. **d** Western blotting of cleaved caspase-3, caspase-9, cleaved PARP, and Bcl-2 analyzed for the indicated times in SH003-treated AGS and SNU-638 cells. **e**–**g** The effect of a pan-caspase inhibitor, Z-VAD-FMK(50 mM, 24 h), on SH003-induced apoptotic cell death. AGS and SNU-638 cells were pretreated with Z-VAD-FMK for 4 h and subsequently treated with SH003 (400 µg/mL, 24 h). Cell viability was determined using the WST-1 assay, and cell cytotoxicity was monitored using the LDH assay; *p < 0.05. Sampling of total lysates was conducted by western blot assay to identify the activation of apoptosis markers such as cleaved caspase-3. β-actin was used as a protein loading control.

To determine the cytotoxic effect of SH003 on various GC cells, we performed the cell viability assay. As shown in Fig. 1a, b, SH003 inhibited the cell viability of these cells in a concentration- and time-dependent manner (0, 100, 200, and 400 μg/mL, 24 h; 0, 8, 16, and 24 h, 400 μg/mL) (Fig. 1a, b). To investigate the cytotoxic effect of SH003, the lactate dehydrogenase (LDH) assay also was performed at various time points (0, 8, 16, and 24 h). As shown in Fig. 1c, the LDH release was significantly enhanced in SH003 (400 µg/mL, 24 h)-treated AGS, SNU-638, and MKN-74 cells. In addition, we examined whether SH003 was associated with caspase-dependent cell death using Western blotting. SH003 treatment significantly increased the pro-apoptotic factors, including cleaved caspase-3, caspase-9, and PARP at various time points (Fig. 1d). We found that SH003 effectively decreased the expression of Bcl-2 at various time points (Fig. 1d). To identify whether SH003-induced cell death is regulated by a pan-caspase inhibitor (Z-VAD-FMK), we treated the GC cells with SH003 (400 µg/mL, 24 h) and Z-VAD-FMK (50 µM, 24 h). This result indicates that Z-VAD-FMK inhibits the decrease of cell viability and the increase of LDH release in SH003-treated GC cells (Fig. 1e, f). Western blotting demonstrates that Z-VAD-FMK plus SH003 decreases the levels of cleaved caspase-3 (Fig. 1g).

### SH003 induces autophagy activation in GC cells

**Fig. 2:**
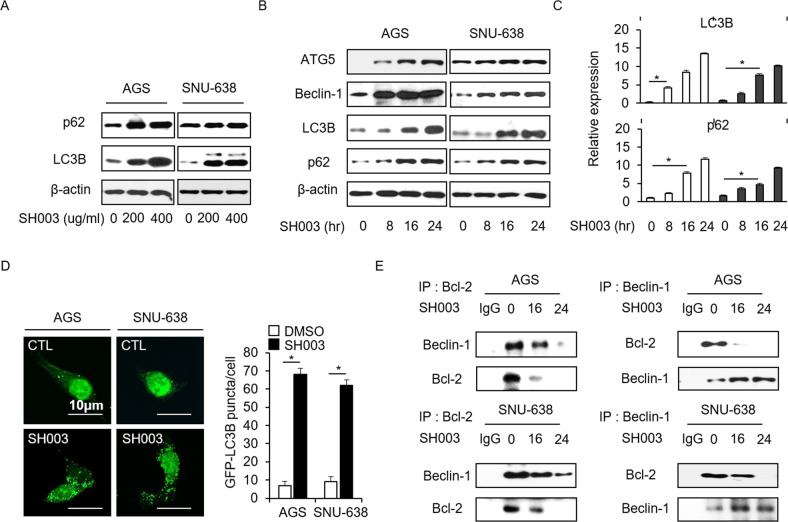
SH003 induces autophagy in GC cells. **a** Western blot analysis of the conversion of LC3-I to LC3-II in SH003-treated AGS and SNU-638 cells treated with SH003 in a dose-dependent manner (0, 200, and 400 µg/mL, 24 h). **b** Western blot analysis of the ATG5, Beclin-1, LC3B, and p62 protein levels in AGS and SNU-638 cells treated with SH003 (400 µg/mL) for the indicated times. **c** Real-time RT-PCR analysis of the LC3B and p62 mRNA levels in AGS cells treated with SH003 (400 µg/mL) for the indicated times. **d** AGS and SNU-638 cells were transfected using the pEGFP-LC3 vector were treated with SH003 (400 µg/mL) for 8 h and analyzed as described in the material and methods section. Fluorescence microscopy analysis calculated by the puncta of GFP-LC3B; *p < 0.05. (E) AGS and SNU-638 cells were treated with SH003 (400 µg/mL) for the indicated times. Bcl-2 was immunoprecipitated in AGS and SNU-638 cells, and the immunoprecipitated proteins were subjected to Western blotting. On immunoprecipitation, Beclin-1 was detected in immunoprecipitates prepared with the anti-Bcl-2 antibody. Bcl-2 was also detected in immunoprecipitates prepared with anti-Beclin-1 antibody by immumoprecipitation.

To examine whether SH003 induces the autophagy processing of LC3-I to LC3-II in SH003-treated GC cells, Western blot assay was performed with autophagy-related proteins, such as LC3B and p62. Consequently, we monitored the accumulation of LC3-II and p62 levels at various dose points. (Fig. 2a). As indicated in Fig. 2a, we identified autophagy activation with various doses of SH003 (0, 200, and 400 µg/mL, 24 h), and SH003 (400 µg/mL) was used to study autophagy activation at various time points. To confirm whether SH003 regulates autophagy, we tested the expression of autophagy markers, such as ATG5, Beclin-1, LC3B, and p62. As expected, SH003 treatment causes cell death by inducing the mRNA and protein levels of ATG5, Beclin-1, LC3B, and p62 at various time points (Fig. 2b, c). Thus, we suggest that autophagy regulates cell death in SH003-treated GC cells. To further identify whether SH003 induces autophagy in GC cells, pEGFP–LC3 vector was transiently transfected into both AGS and SNU-638 cells. DMSO-treated cells have weak LC3 puncta, whereas SH003-treated cells exhibit increasing green LC3 puncta in the cytoplasm (Fig. 2d). Bcl-2 is an anti-autophagy protein that functions via inhibitory interaction with Beclin-1, and Bcl-2/Beclin-1 complex formation is an important step in the autophagy process^21^. To identify whether SH003 treatment regulates Bcl-2 expression in GC cells and whether the interaction between Bcl-2 and Beclin-1 dissociates via autophagy activation, co-immunoprecipitation was carried out using antibodies Bcl-2 and Beclin-1. Consequently, it was observed that the incubation of GC cell lysates with SH003 decreased the Bcl-2 level in the Beclin-1-immunoprecipitated fractions and reduced the Beclin-1 level in the Bcl-2-immunoprecipitated fractions at various time points (Fig. 2e). Therefore, SH003 induces the dissociation of the Bcl-2/Beclin-1 complex in GC cells.

### Autophagy inhibition regulates SH003-induced cell death

**Fig. 3:**
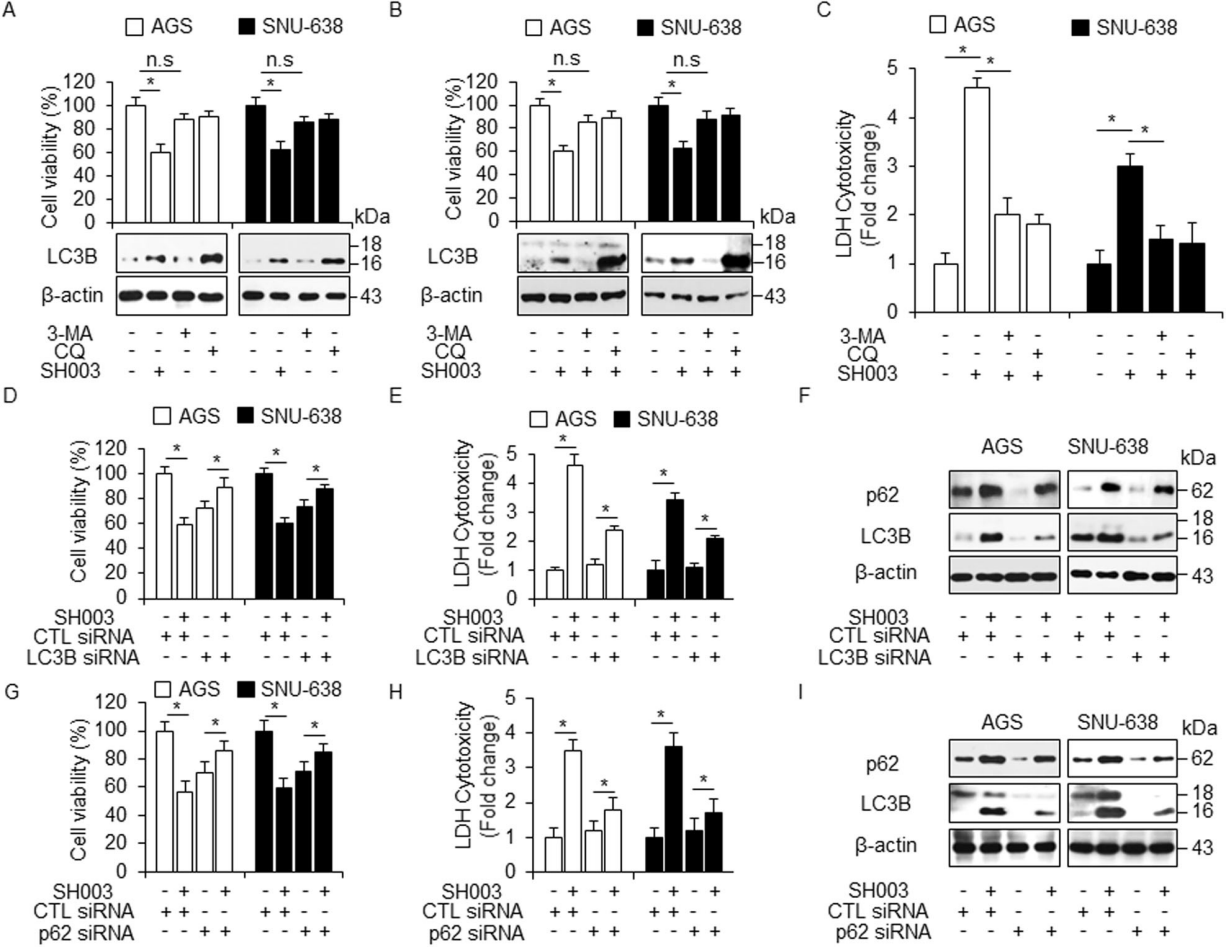
Autophagy inhibition induces cell survival flux in SH003-treated GC cells. **a** Cell viability using WST-1 assay and western blot analysis of LC3B in AGS and SNU-638 cells treated with SH003 (400 µg/mL) or 3-MA (5 mM) or CQ (20 μM) for 24 h; *p < 0.05. **b**, **c** Cell viability, LDH assay, and western blot analysis of LC3B in SH003 alone, SH003 + 3-MA (5 mM), and SH003 + CQ (20 μM)-treated AGS and SNU-638 cells for 24 h; *, *p* < 0.05. (D*-*I) After AGS and SNU-638 cells were transfected by LC3B and p62 siRNAs in AGS and SNU-638 cells, Cell viability, LDH activity, and Western blot analyses with SH003 (400 µg/mL, 24 h) treatment were performed; *p < 0.05. β-actin was used as a protein loading control.

To clarify the relation between SH003-induced autophagy and cell death in GC cells, we examined the effect of 3-methyladenine (3-MA) and chloroquine (CQ), an inhibitor of autophagy, on cell viability. 3-MA and CQ did not affect cell viability in GC cells; the 3-MA treatment reduced the activation of LC3B, whereas the CQ treatment further increased the induction of LC3-II (Fig. 3a). To investigate whether the activation of LC3B correlated with increased autophagic flux during SH003 treatment, GC cells were treated with SH003 in the presence or absence of 3-MA or CQ. 3-MA or CQ in combination with SH003 did not affect the cell viability in GC cells (Fig. 3b). In line with this evidence, 3-MA reduced the activation of LC3B in SH003-treated GC cells, whereas CQ increased the accumulation of LC3-II (Fig. 3b). Furthermore, the LDH release was significantly inhibited in SH003 + autophagy inhibitor-treated GC cells (Fig. 3c). To measure the number of autophagosome in SH003-treated GC cells, we stained GC cells using Cyto-ID dye and performed FACs analysis. BafA1 or CQ in combination with SH003 in AGS cells mediated higher Cyto-ID signal compared with BafA1 or CQ alone, and Western blotting analysis also induces the accumulation of LC3-II with BafA1 or CQ treatment in SH003-treated AGS and SNU-638 cells (Supplementary Fig. 1A, B). Thus, SH003 treatment with autophagy inhibitors did not affected the cell viability and instead supported survival by inhibiting autophagy. The above pharmacological findings suggest that SH003 treatment induces autophagic cell death in GC cells, and SH003 + autophagy inhibitor treatment significantly inhibits cell death. To further identify whether SH003 regulates autophagic cell death in GC cells, we used to two specific siRNAs, namely LC3B and p62. GC cells were transfected with LC3B and p62 siRNAs. The cell viability in SH003-treated LC3B and p62 knockdown cells was significantly enhanced compared with that in control siRNA-transfected cells; conversely, LDH release was significantly reduced in SH003-treated LC3B and p62 knockdown cells compared with that in control siRNA-transfected cells (Fig. 3d, e, g, h). After LC3B and p62 were depleted by knockdown experiments, LC3B and p62 levels were analyzed by Western blotting. As shown in Fig. 3f, i, and Supplementary Fig. 1C, unlike control siRNA transfection, LC3B, and p62 depletion blocked the accumulation of LC3-II and p62 levels of in SH003-treated GC cells. Therefore, our evidences suggest that autophagy inhibition plays a survival role in SH003-treated GC cells.

**Fig. 4:**
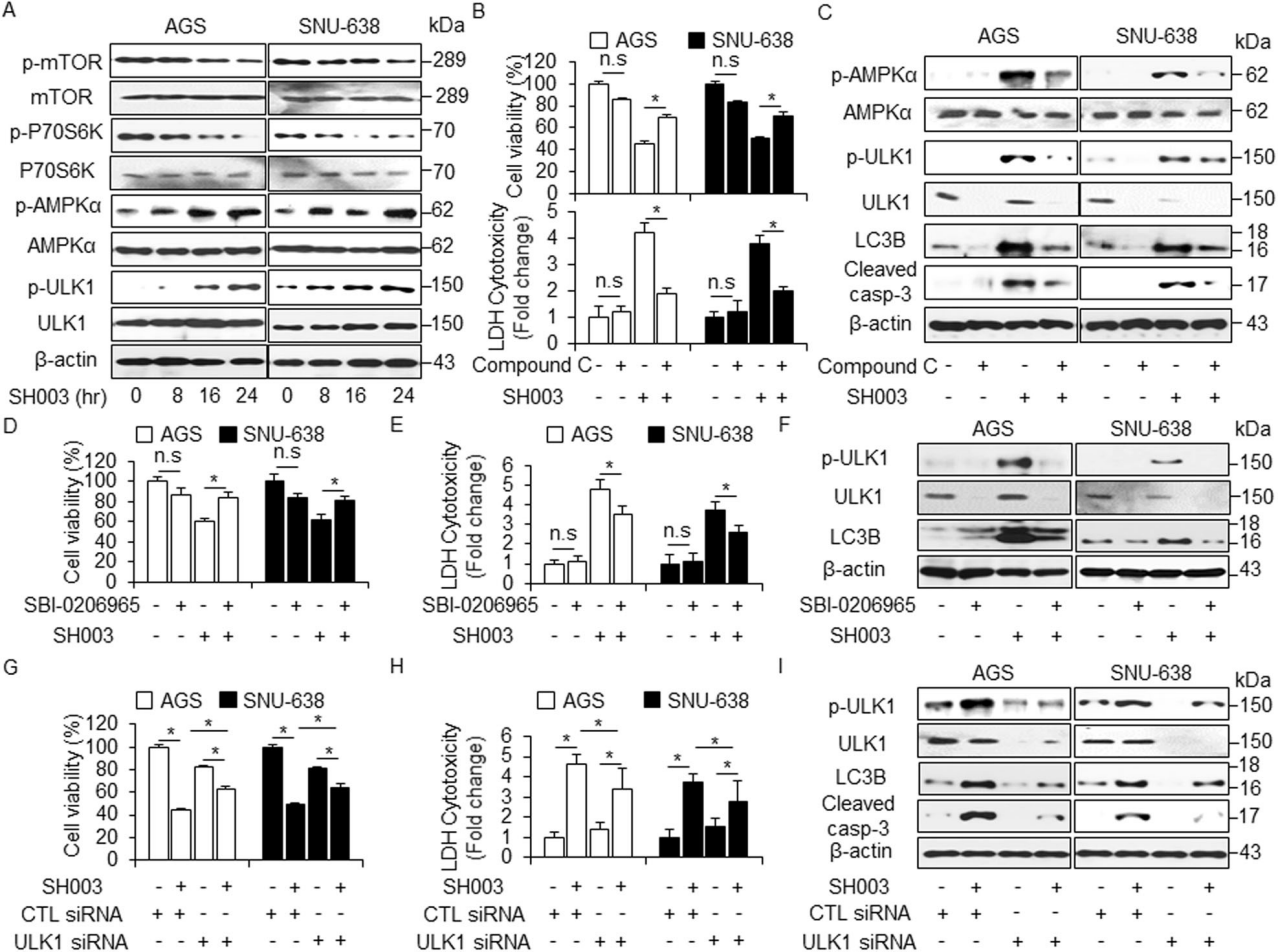
Inhibition of AMPKα/ULK1 mediates cell survival in SH003-treated GC cells. **a** AGS and SNU-638 cells were treated with SH003 (400 μg/mL) for the indicated times. After SH003 treatment, cell lysates were loaded for Western blotting and detected antibodies targeting p-AMPKα (Thr172), AMPKα, p-ULK1 (Ser555), ULK1, p-mTOR (Ser2448), and p-p70S6K (Thr389) for autophagy induction. β-actin was used as a protein loading control. **b**, **c** Cell viability, LDH release, and Western blot analyses of p-AMPKα, p-ULK1, LC3B, and cleaved caspase-3 in the AGS and SNU-638 cells treated with SH003 (400 μg/mL, 24 h) in the presence or absence of Compound C (2 μM, 24 h); *p < 0.05. (D-F) Cell viability, LDH release, and Western blot analysis of p-ULK1 and LC3B in the AGS and SNU-638 cells treated with SH003 (400 μg/mL, 24 h) in the presence or absence of SBI-0206965 (10 μM, 24 h); *p < 0.05. (G*-*I) Cell viability, LDH release and western blot analyses of p-ULK1, LC3B, and cleaved caspase-3 in the AGS and SNU-638 cells treated with SH003 (400 μg/mL, 24 h) in the presence or absence of ULK1 siRNA (30 nM, 24 h); *p < 0.05. β-actin was used as a protein loading control.

The effects of SH003 on the mTOR/AMPKα/ULK1 pathway were examined by Western blot and the results are shown in Fig. 4a. In GC cells, SH003 treatment time-dependently decreased the levels of p-mTOR and p-P70S6K and enhanced the levels of p-AMPKα and p-ULK1 (Fig. 4a). To determine the role of AMPKα in SH003-induced autophagic cell death, Compound C, a specific AMPK inhibitor, was used to inhibit AMPKα phosphorylation and to assess the level of autophagy. As expected, Compound C inhibited SH003-mediated cell death, which was indicated by an increase in cell viability and a decrease in the LDH release in the SH003 + Compound C-treated GC cells compared with control cells (Fig. 4b). The inhibition of AMPKα resulted in the reduced expression of p-AMPKα, p-ULK1, cleaved caspase-3, and LC3-II in the SH003 plus Compound C-treated GC cells than in SH003-treated GC cells (Fig. 4c). These findings suggest that SH003 inhibits the activation of the mTOR pathway and activates the AMPK/ULK1 pathway in GC cells. Growing evidences indicate that ULK1 plays an essential function on autophagy activation and recruit ATG proteins (ATGs) of downstream to autophagosome formation^22^. To study whether ULK1 regulates autophagy process during SH003 treatment, we utilized SH003 with or without of SBI-0206965 (SBI) as ULK1 inhibitor. SBI inhibited SH003-mediated cell death; this was indicated by an increase in cell viability and a decrease in the LDH release in the SH003 + SBI in GC cells compared with control cells (Fig. 4d, e). As demonstrated, SBI dramatically down-regulated ULK1 in GC cells, and SBI plus SH003 reduced the expression levels of p-ULK1 and LC3-II compared to SH003 alone (Fig. 4f). In accordance with our findings, ULK1 inhibition blocked SH003-initiated autophagy activation in GC cells, indicating that ULK1 activation is important for SH003-mediated autophagy induction. To further clarify the mechanisms involved in SH003-induced autophagic cell death in GC cells, ULK1 knockdown experiment performed. Unlike control siRNA-transfected cells, ULK1 siRNA-mediated silencing reduced the abilities of SH003 to suppress cell viability and to mediate the LDH release in SH003-treated GC cells (Fig. 4g, h). ULK1 knockdown effectively suppressed the SH003-induced ULK1 phosphorylation, LC3-II, and cleaved caspase-3 (Fig. 4i). Together, these findings reveal that the AMPK/ULK1/LC3B pathway plays an important role in mediating the anti-cancer effects of SH003 in GC cells.

### SH003 induced cell death via PERK signaling of UPR in GC

**Fig. 5:**
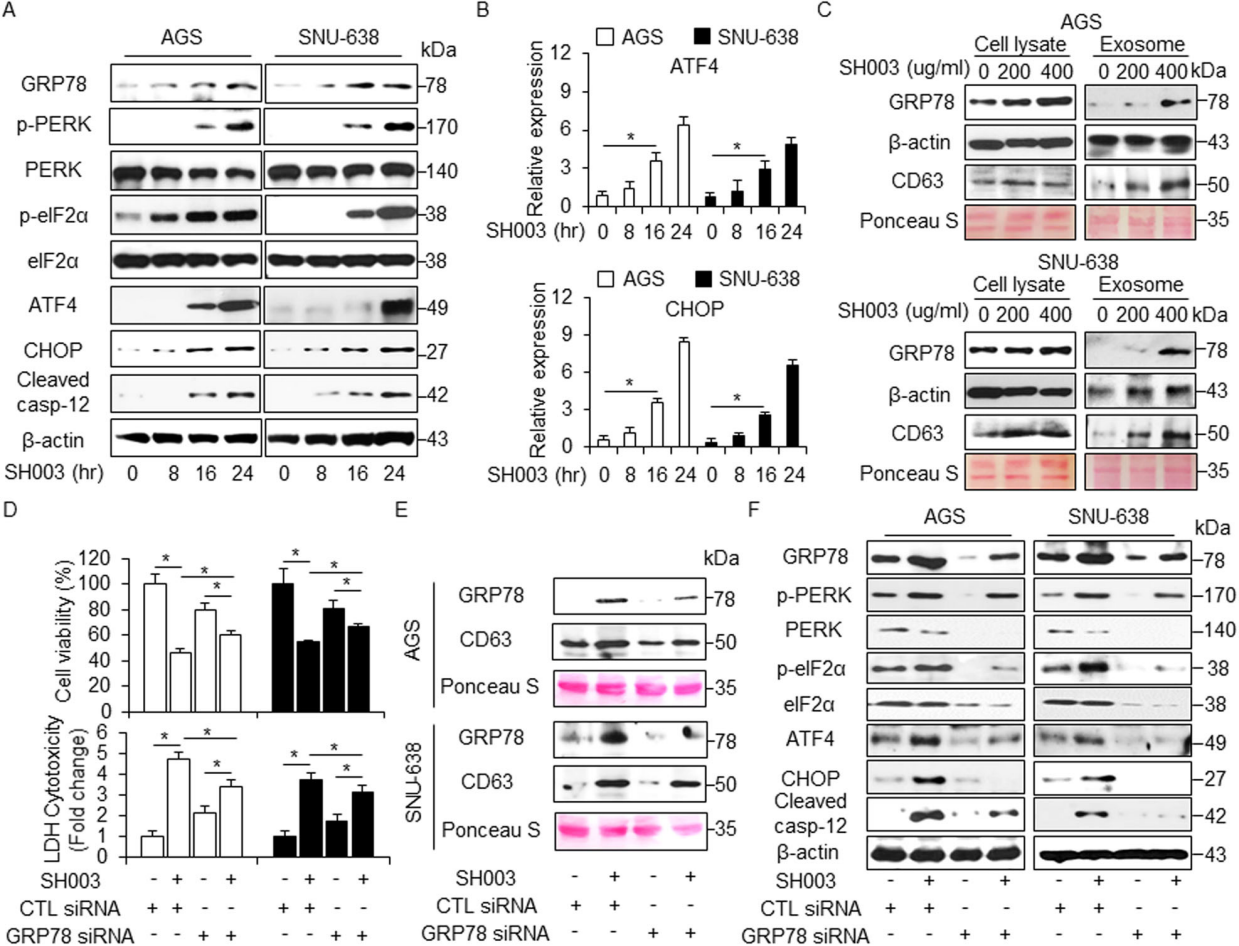
SH003 induces ER stress response in GC cells. **a** AGS and SNU-638 cells were treated with SH003 (400 μg/mL) for indicated times and ER stress markers including GRP78, p-PERK, PERK, p-eIF2α, eIF2α, ATF4, CHOP, and cleaved caspase-12 were assessed by Western blot assay. **b** AGS and SNU-638 cells were treated with SH003 (400 μg/mL) for the indicated times and ER stress markers, including ATF4 and CHOP, were assessed by real-time RT-PCR. **c** AGS and SNU-638 cells were treated with SH003 in a dose-dependent manner (0, 200, and 400 μg/mL, 24 h) and exosomes were collected from the supernatant of the cells. Protein purification for exosomes and cell lysates was quantified by Ponceau S staining. These samples were also examined by Western blotting using the exosome marker, CD63 and the ER stress marker, GRP78. **d** AGS and SNU-638 cells were transfected with control or GRP78 siRNA in the presence or absence of SH003 (400 μg/mL, 24 h), and then, cell viability and LDH assay were performed; *p < 0.05. **e** Western blot analysis of GRP78 and CD63 in exosomes isolated from SH003 (400 μg/mL, 24 h)-treated AGS and SNU-638 cell culture media in the presence or absence of GRP78 siRNA (30 nM, 24 h).). β-actin and Ponceau S were used as protein loading controls. **f** Western blot analysis of GRP78, p-PERK, p-eIF2α, ATF4, CHOP, and cleaved caspase-12 in SH003 (400 μg/mL, 24 h)-treated AGS and SNU-638 cells in the presence or absence of GRP78 siRNA (30 nM, 24 h). β-actin was used as protein loading controls.

Increasing evidence suggests that ER stress plays an important role in autophagy and cell death^23^. To identify whether SH003 regulates the ER stress pathway in GC cells, we investigated the changes in the levels of ER stress-related proteins, such as GRP78, p-IRE1α, IRE1α, p-JNK, JNK, p-PERK, PERK, p-eIF2α, eIF2α, ATF4, cleaved caspase-12, and CHOP using Western blotting in a time-dependent manner. SH003 leads to a time-dependent increase in the levels of ER-stress markers (Fig. 5a and Supplementary Fig. 2A). Furthermore, mRNA levels of ATF4 and CHOP were markedly increased in SH003-treated GC cells (Fig. 5b).

**Fig. 6:**
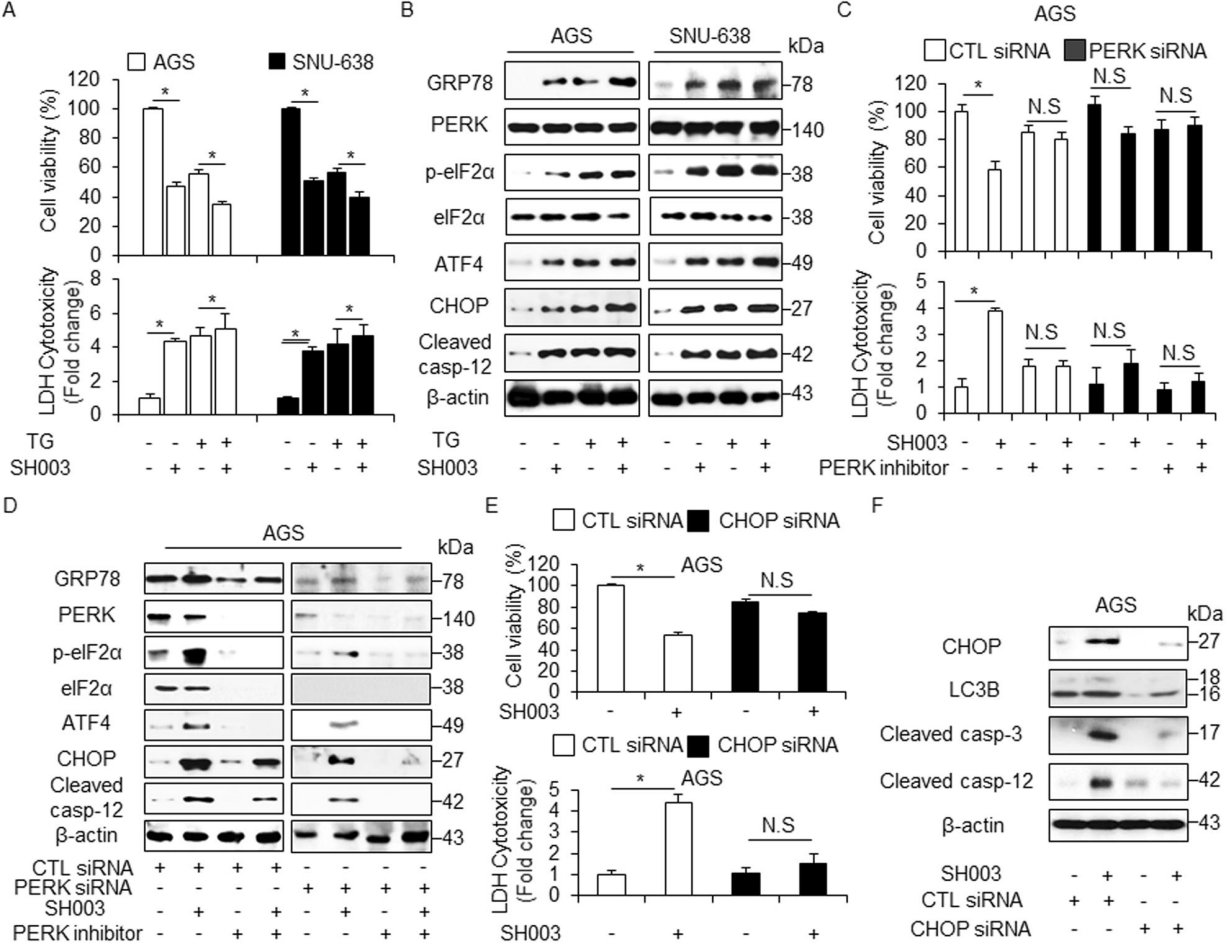
Induction and inhibition of ER stress response regulate SH003-induced autophagic cell death in GC cells. **a**, **b** Cell viability, LDH release, and western blotting of in GRP78, p-eIF2α, ATF4, CHOP, and cleaved caspase-12 levels were determined using WST-1 and LDH assays in thapsigargin (3 μM, 24 h) and SH003 (400 μg/mL, 24 h)-treated GC cells; *p < 0.05. **c**, **d** Cell viability, LDH release, and Western blot analyses of GRP78, PERK, p-eIF2α, ATF4, CHOP, and cleaved caspase-12 in the AGS cells treated with SH003 (400 μg/mL, 24 h) were performed in the presence or absence of a PERK inhibitor (10 μM, 24 h); *p < 0.05. **e**, **f** Cell viability, LDH release, and Western blot analyses of CHOP, LC3B, cleaved caspase-3, and cleaved caspase-12 in the AGS cells treated with SH003 (400 μg/mL, 24 h) were performed in the presence or absence of CHOP siRNA (30 nM, 24 h); *p < 0.05. β-actin was used as a protein loading control.

A recent report suggests that the ER chaperone GRP78/Bib is released into the extracellular space via exosomes in cancer cells^24^. To analyze the role of GRP78 on SH003-induced exosome production, we treated GC cells with SH003 and purified the secreted exosomes from their culture supernatants. SH003 treatment increased the secretion of the exosome marker CD63 in a dose-dependent manner, and GRP78 expression was significantly higher in exosomes derived from SH003-treated cell culture medium compare to DMSO (Fig. 5c). These results suggest that the exosomal GRP78 contributes to SH003-induced autophagic cell death. To further identify the exosomal GRP78 involved in SH003-induced autophagic cell death in GC cells, GRP78 knockdown study performed. GRP78 siRNA-mediated silencing induced the increase of cell viability and the decrease of LDH release in SH003-treated GC cells compare to control cells (Fig. 5d). Furthermore, exosomes on GRP78 knockdown GC cells were decreased by SH003, indicating that SH003-induced ER stress contributes to the increased exosome release (Fig. 5e). Compare to controls with SH003 treatment, GRP78 inhibition with SH003 treatment resulted in an inhibition of the p-PERK, p-eIF2α, ATF4, CHOP, and cleaved caspase-12. (Fig. 5f). We investigated the effect of thapsigargin (TG), an ER stress inducer, in combination with SH003 on GC cells. Combination experiments showed that TG + SH003 decreased cell viability and increased LDH release compared with control conditions (Fig. 6a). Furthermore, we observed an increase in GRP78, p-eIF2α, ATF4, CHOP, and cleaved caspase-12 levels (Fig. 6b and Supplementary Fig. 2B).

### ER stress inhibition blocks SH003-induced autophagy activation

We performed PERK knockdown experiments in SH003-treated GC cells. These cells were transfected with PERK siRNA (30 nM, 24 h) and treated with SH003 + PERK inhibitor (10 μM). In control siRNA-transfected GC cells, SH003 reduced cell viability and increased cell death, whereas combination treatment of SH003 and a PERK inhibitor more increased cell viability and decreased cell death to a greater extent than observed with SH003 treatment (Fig. 6c and Supplementary Fig. 3A, B). Interestingly, in PERK knockdown GC cells, both SH003 and SH003 + PERK inhibitor did not affect cell viability and LDH release (Fig. 6c and Supplementary Fig. 3A, B). Western blotting revealed that negative control siRNA-transfected GC cells enhanced the expression of GRP78, p-eIF2α, ATF4, CHOP, and cleaved caspase-12 with SH003 treatment, whereas SH003 plus PERK inhibitor reduced the expression of these ER stress-related markers (Fig. 6d and Supplementary Fig. 3C). ER stress markers had lower expression in PERK knockdown GC cells than in control siRNA-transfected GC cells. Furthermore, using cell viability and the LDH assay, it was found that CHOP knockdown largely prevented to SH003-induced autophagic cell death in GC cells compared with control cells (Fig. 6e and Supplementary Fig. 3D, E). CHOP inhibition + SH003 treatment led to the down-regulation of LC3B, CHOP, cleaved caspase-3, and cleaved caspase-12 in GC cells unlike in control cells (Fig. 6f and Supplementary Fig. 3F). To identify the effect of IRE1α-JNK axis in SH003-treated ER stress, we treated with JNK inhibitor SP600125 in SH003-treated AGS cells and performed cell viability assay and Western blotting analysis. SP600125 inhibited the decrease of cell viability in SH003-mediated AGS cells and blocked the increase of p62, LC3B, and cleaved caspase-3 (Supplementary Fig. 4A, B). Therefore, SH003 regulates autophagic cell death via ER stress in GC cells.

### SH003 induces more autophagic cell death in hypoxia than in normoxia

**Fig. 7:**
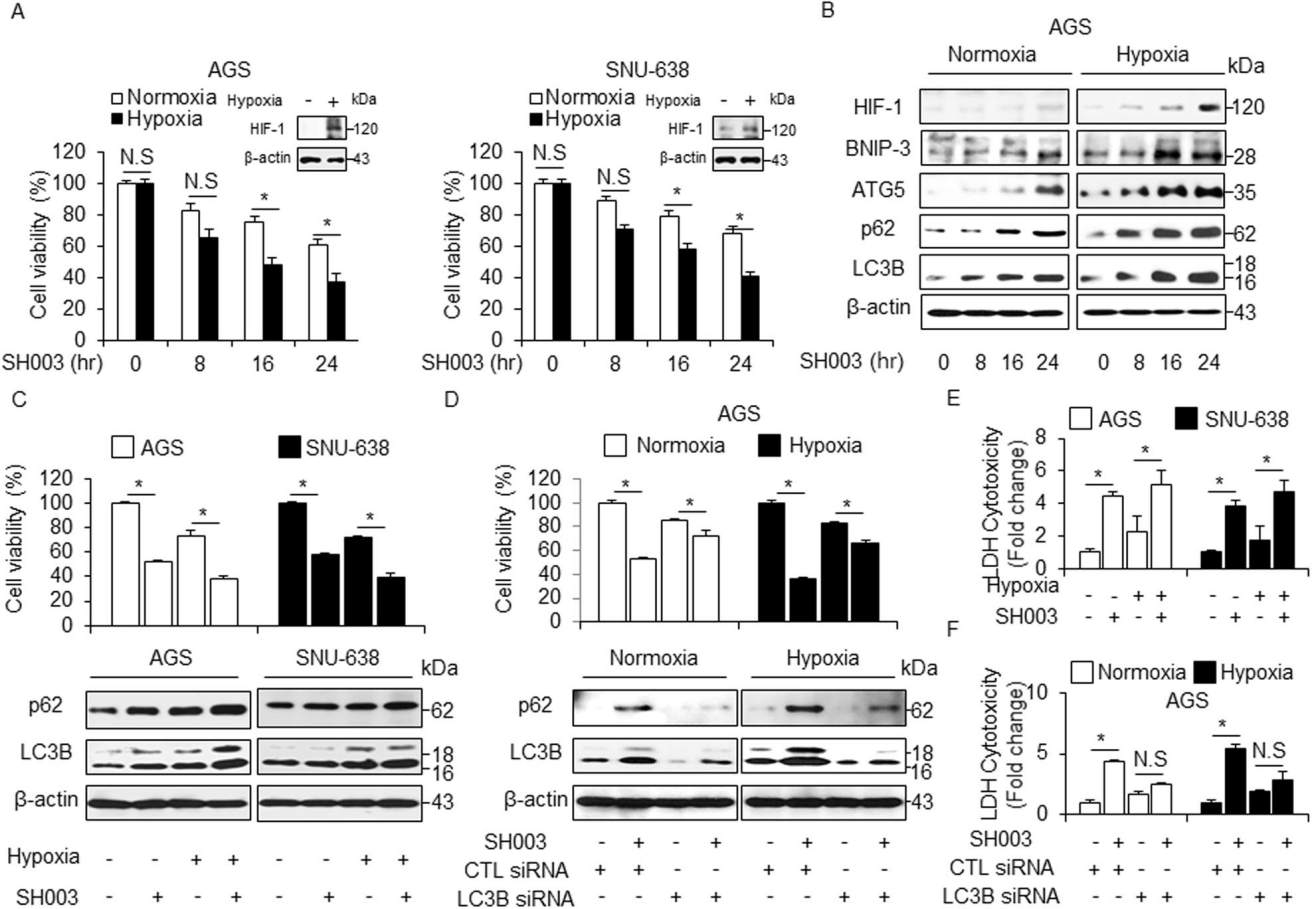
SH003 induces more autophagic cell death in hypoxia than in normoxia. **a** SH003 (400 μg/mL) was added to AGS and SNU-638 cells in a time-dependent manner. Cultures were also exposed to hypoxia or normoxia in a time-dependent manner. Cell viability was assessed using WST-1 assay; *p < 0.05. Western blot analysis was used to validate the targeted changes in HIF-1α expression in hypoxia-induced AGS and SNU-638 cells. **b** Expression of HIF-1α, BNIP3, ATG5, p62, and LC3B in SH003 (400 μg/mL, 24 h)-treated AGS cells for the indicated times under normoxia or hypoxia. **c**, **e** Cell viability analyses, LDH assay, and Western blotting in hypoxia-mediated AGS and SNU-638 cells were analyzed in the presence of SH003 (400 μg/mL, 24 h). β-actin was used as a protein loading control. **d**, **f** AGS cells were transfected with control or LC3B siRNA and then exposed to normoxia or hypoxia for 24 h in the presence of SH003 (400 μg/mL, 24 h). Cell viability analyses, LDH assay, and Western blotting were performed for this condition.

Hypoxia is a general phenomenon of tumor environment and many cancer patients with severely hypoxic tumor have lower survival rates than less hypoxic tumor^25^. Contrastively, a recent report suggests that hypoxia induces cell death via autophagy activation in cancer cells^26^. Furthermore, hypoxia often induces autophagic cell death via induction of HIF-1 and BNIP3 in cancer^27^. GC cells exhibited a time-dependent inhibition of cell viability in response to SH003 in both normoxia and hypoxia, with significantly inhibited cell viability under hypoxia (Fig. 7a). To identify whether SH003 shows differential regulation in normoxia- and hypoxia-induced GC cells, we performed Western blotting. HIF-1α, BNIP3, ATG5, p62, and LC3B were found to be expressed more in hypoxia + SH003-induced GC cells than in normoxia + SH003-mediated cells (Fig. 7b and Supplementary Fig. 5A). Furthermore, SH003 treatment induces cell viability inhibition, the LDH release, and autophagy activation in hypoxia-induced GC cells unlike in SH003-mediated GC cells or only hypoxia-induced GC cells (Fig. 7c, e). To determine SH003-induced autophagic cell death in hypoxia- and normoxia-induced LC3B knockdown GC cells, we evaluated the cell viability and LDH release in LC3B knockdown GC cells. SH003 caused a greater reduction of cell viability in the hypoxia-induced GC cells than in normoxia-induced GC cells (Fig. 7d and Supplementary Fig. 5B). LC3B knockdown markedly inhibited SH003-induced autophagic cell death in GC cells subjected to normoxia and hypoxia, as determined by a decrease in LC3-II and p62 (Fig. 7d and Supplementary Fig. 5C). Further, we found that the amounts of LC3-II and p62 proteins were increased by a greater extent in hypoxic GC cells than in normoxic GC cells (Fig. 7d and Supplementary Fig. 5C). The SH003-mediated LDH release was slightly higher in hypoxia-induced LC3B knockdown cells than in the normoxia-induced GC cells (Fig. 7f and Supplementary Fig. 5D). These results indicate that SH003 induces higher autophagic cell death in hypoxia-mediated GC cells.

### SH003-induced autophagy is regulated via STAT3-G9a axis

**Fig. 8:**
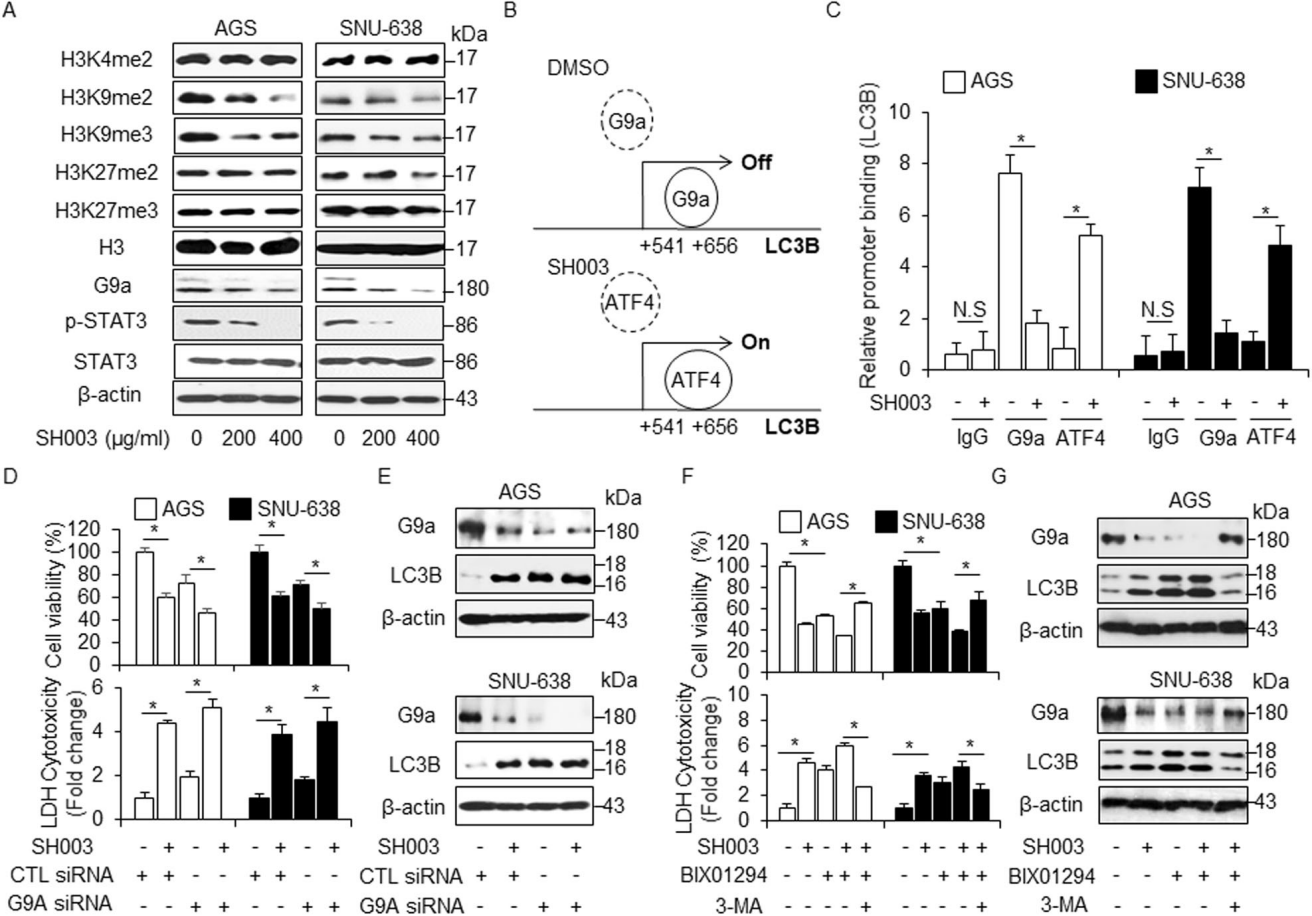
SH003-induced autophagy is regulated via G9a. **a** Protein levels of H3K4me2, H3K9me2, H3K9me3, H3K27me2, and H3K27me3 were investigated by Western blotting in nuclear fraction samples isolated from SH003 (0, 200, and 400 μg/mL, 24 h)-treated AGS and SNU-638 cells, and H3 was used as a protein loading control. Protein levels of G9a and p-STAT3 were detected by Western blot assay in SH003-treated AGS and SNU-638 cells and β-actin was used as a protein loading control. **b**, **c** The localization of the G9a and ATF4 on the *LC3B* promoter. SH003 regulates G9a and ATF4 binding at the *LC3B* promoter. SH003 (400 μg/Ml, 24 h) treatment was performed in AGS and SNU-638 cells, and real-time ChIP assays of the *LC3B* promoter region (+541 to +656) were performed with G9a and ATF4 antibody. **d**, **e** AGS and SNU-638 cells were transfected with control or G9a siRNA in the presence or absence of SH003 (400 μg/mL, 24 h), and then, cell viability tests, LDH assay, and Western blotting were performed; *p < 0.05. **f**, **g** AGS and SNU-638 cells were pretreated with 3-MA (5 mM) for 4 h, and then, were treated with SH003 (400 μg/mL, 24 h) and/or BIX-01294(10 μM, 24 h). Cell viability, LDH release, and Western blot analyses of G9a and LC3B in the AGS and SNU-638 cells treated with SH003 + BIX-01294 + 3-MA were performed. β-actin was used as a protein loading control; *p < 0.05.

EHMT2/G9a causes dimethylation of histone H3 and down-regulates LC3B, whereas G9a inhibition induces autophagy activation by inhibiting H3K9me2^28^. STAT3 interacts with G9a and STAT3-G9a-mediated epigenetic silencing promotes cancer progression^29^. Furthermore, our previous report suggests that SH003 inhibits STAT3 activation and induces cell death in cancer cells^12,30^. We observed a dose-dependent decrease of H3K9me2 and H3K9me3 levels in response to SH003 treatment, whereas this treatment did not affect H3K4me2, H3K27me2, and H3K27me3 levels (Fig. 8a). Notably, SH003 resulted in decreased levels of p-STAT3 and G9a in a dose-dependent manner (Fig. 8a). Recent reports suggest that severe hypoxia induces ER stress via PERK-ATF4 axis and ATF4 binding on the *LC3B* promoter (+541~+656) mediates autophagy breast cancer cells, whereas G9a directly binds on *LC3B* promoter and regulates repressively *LC3B* expression, and G9a inhibition activates *LC3B* expression and autophagy^31,32^. On the based on these report, to identify the candidate regulators, such as G9a and ATF4, on the *LC3B* promoter (+541 to +656), we performed quantitative chromatin immunoprecipitation (qChIP) to identify the G9a and ATF4 binding on the *LC3B* promoter in GC cells (Fig. 8b, c). Chromatin samples from GC cells grown under SH003 treatment were immunoprecipitated with G9a and ATF4 antibody (Fig. 8c). This experiment suggested that G9a binds on the *LC3B* promoter in DMSO-treated GC cells but not ATF4. However, SH003 treatment inhibits G9a binding and induces ATF4 binding on the *LC3B* promoter. Together, these findings indicate that G9a binding on the *LC3B* promoter represses autophagy process, but SH003 induces autophagic cell death via suppression of G9a and binding of ATF4. To examine whether SH003-mediated STAT3-G9a inhibition and subsequent decreases in H3K9me2 are related to SH003-induced autophagic cell death, we performed cell viability, LDH cytotoxicity, and Western blot assay in G9a knockdown GC cells. SH003 treatment induces decreased cell viability and increased LDH release and LC3-II accumulation in G9a knockdown GC cells but not in control GC cells (Fig. 8d, e). These results indicate that SH003 induces autophagic cell death by inhibiting STAT3-G9A axis in GC cells. BIX-01294 (BIX) is also known as selective inhibitor for G9a and inducer of autophagic cell death in various cancer types^33^. As shown in Fig. 8e–g, BIX decreased cell viability and increased the LDH release and LC3-II levels in SH003-treated GC cells. In contrast, 3-MA treatment mediated an increase of cell viability and G9a expression and a decrease of the LDH release and LC3-II level in BIX plus SH003-treated cells (Fig. 8f, g). To further probe STAT3-G9a axis in SH003-treated GC cells, we performed ChIP assay for the STAT3 binding on *G9a* promoter and SH003 dissociated the binding of STAT3 and G9a (Supplementary Fig. 6A, B). Moreover, STAT3 knockdown was down-regulated G9a expression in SH003-treated AGS cells (Supplementary Fig. 6C). These finding suggest that 3-MA suppresses SH003 and BIX-induced autophagic cell death. Therefore, SH003 induces autophagic cell death by inhibiting the STAT3-G9a pathway in GC cells.

## Discussion

In the present report, we found that SH003 induces autophagic cell death via ER stress (PERK/ATF4/CHOP) in GC cells. In addition, hypoxia mediates autophagic cell death by inducing of HIF-1 and BNIP3 in SH003-treated GC cells. Autophagic vacuoles of autophagosomes were observed in SH003-treated GC cells. We demonstrated that targeting ER stress and autophagy could suppress SH003-induced autophagic cell death. Adaptation to ER stress depends on the activation of the unfolded protein response (UPR) and protein degradation signaling such as autophagy, thereby promoting cell survival and adaptation^34^. However, under chronic or irreversible ER stress responses, the UPR (PERK, ATF6, and IRE1α) promotes autophagic cell death and several Bcl-2 family proteins play an important role in autophagic cell death^35^. Furthermore, PERK–eIF2α–CHOP signaling contributes to autophagy and apoptosis in several cancer types^36^. Our data suggest that SH003-induced PERK–eIF2α–ATF4–CHOP axis contributes to autophagic cell death in GC cells. The ER chaperone GRP78 is a key regulator contributing to autophagy activation^37^. Tunicamycin increases ATF4 and CHOP activity via GRP78 up-regulation in various cancer types and induces autophagy and apoptosis^38^. GRP78 regulates autophagy and cell death by modulating the mTOR–AMPK pathway in cancer^39^. To identify the potential mechanisms by which GRP78 induces autophagic cell death in SH003-treated GC cells, we investigated the mTOR–AMPK–ULK1 pathway under GRP78 induction. Our results showed that the phosphorylation of AMPK and ULK1 was enhanced in SH003-treated GC cells, whereas that of mTOR and p70S6K was reduced. Accordingly, AMPK and ULK1 inhibition attenuated autophagy and cell death, indicating that SH003 induces autophagic cell death by activating AMPK and ULK1. A recent report suggested that GRP78 inhibition in ovarian cancer cells blocked ER stress and autophagy activation induced by diindolylmethane and inhibited AMPK via mTOR activation^40^. Furthermore, increasing evidence suggests that GRP78 is secreted from cancer cells via exosomes and that GRP78 secretion was increased by HDAC inhibitors^24,41^. Our finding suggests that SH003 induces ER stress by accumulating cell lysate and exosomal GRP78 and mediates autophagic cell death by controlling the mTOR–AMPK–ULK1 pathway. However, targeting GRP78 reduces SH003-mediated autophagic cell death and exosome release. CHOP is also involved in ER stress and autophagic cell death^42^. Our data indicates that CHOP knockdown prevents SH003-induced autophagic cell death in GC cells. Similarly, PERK inhibition reverses the effect of SH003 on cell viability, LDH release, and autophagy activation, suggesting that the SH003-mediated mechanism is dependent on PERK signaling dependent. Autophagy is a dynamic cellular process of the lysosomal degradation of cellular components after multiple forms of cellular stress, such as ER stress, protein aggregation, and organelle damage^43^. Recent reports have described that prolonged ER stress and autophagy activation may eventually lead to apoptosis and cell death and called it type II cell death^44^. Interestingly, we observed that autophagy inhibition restored SH003-treated cell survival. Under SH003-mediated ER stress, PERK activation increases the expression of LC3B, p62, and ATG5 through ATF4 and CHOP activation. Therefore, PERK is important for SH003-induced autophagic cell death via ER stress. Accumulating reports suggest that BIX-01294, a G9a inhibitor, induces autophagic cell death via ER stress in various cancer types^45^. In this study, genetic analysis of the *LC3B* promoter indicated consensus binding for the G9a and ATF4 transcription factors using ChIP. We hypothesized that differential binding of G9a and ATF4 might regulate LC3B expression and autophagy during SH003 treatment. ChIP assay revealed that G9a was induced to bind the *LC3B* promoter during DMSO treatment, but ATF4 was mediated to bind the *LC3B* promoter after stimulation by SH003 treatment and G9a binding suppressed by ATF4 binding. Taken together, we show that G9a is bound and acts as a repressor at the *LC3B* promoter during DMSO treatment and SH003 treatment represses the expression of STAT3, H3K9me2, and G9a via PERK/ATF4 signaling and induces ER stress and autophagic cell death. Interestingly, 3-MA blocks SH003-mediated autophagic cell death via G9a inhibition.

Hypoxia is recognized as a major obstacle in chemotherapy, as it may modulate drug response via regulation of Bcl-2 family proteins^46^. Increasing evidence indicates that prolonged hypoxia mediates autophagic cell death by regulating Bcl-2 family proteins and exerts pro-death effects^47^. In this study, SH003 induces autophagic cell death and HIF-1 and BNIP3 dependence to a greater extent under hypoxia conditions than under normoxia conditions and increases the anti-GC effect. Interestingly, autophagy inhibition protects SH003-induced cell death under hypoxia conditions. Therefore, SH003 is potentially significant for the therapy of GC under hypoxia conditions.

In summary, we demonstrated that SH003 inhibits tumor growth through autophagic cell death mechanisms initiated by ER stress and represses STAT3 and G9a. These findings provide important insights into the molecular mechanism of SH003 in GC therapy. Furthermore, our study may establish a novel link between ER stress, epigenetics, and autophagy, which suggests new insights for cancer treatment.

## Materials and methods

### SH003 extraction

SH003 was extracted as previously described^7^. The herbal formula was originally designed for cancer therapy. The three ingredients and their amounts (g) were as follows: 333 g of *Astragalus membranaceus*, 333 g of *Angelica gigas*, and 333 g of *Trichosanthes kirilowii Maximowicz*. The mixtures were obtained by Han-Poong Pharm co. Ltd (Jeonjoo, Republic of Korea). Herbal medicines were mixed together, soaked in 30% ethanol, and extracted by 100 °C treatment for 2 h. The extract was then filtered, evaporated, and lyophilized to make the SH003 powder. This was stored at −80 °C until use.

### Cell culture

The human GC cells were purchased from the Korean Cell Line Bank (Cancer Research Center, Seoul National University, Seoul, Korea). Cells were cultured in RPMI1640 medium (Welgene) supplemented with 5% fetal bovine serum (Gibco) and 100 μg/mL antibiotics (100 U/ mL penicillin and 100 μg/ mL streptomycin, Gibco) in a 5% CO_2_ humidified incubator at 37 °C.

### Cell viability assay

WST-1 assay was performed according to the manufacturer’s instructions (Roche) with 10 μL of WST-1 reagent added to each well of a 96-well plate. After 1 h of incubation using CO_2_ incubator, the conversion of WST-1 reagent into chromogenic formazan was evaluated with a spectrophotometer (Molecular devices).

### LDH assay

AGS and SNU-638 cells were seeded into a 96-well plate with the growth medium. To determine the LDH (Thermo Scientific Pierce) activity in supernatants, 100 μL of the reaction mixture was added, and incubation for 30 min was done in a dark room. The LDH activity measured the absorbance of the samples at 490 or 492 nm using the ELISA reader.

### Transfection

AGS and SNU-638 cells in a six-well plate were transfected with double-stranded siRNAs (30 nmol/mL), including LC3B (Bioneer), ULK1(Bioneer), GRP78 (Bioneer), CHOP (Bioneer), G9a (Santacruz), and p62 (CellSignaling), for 24 h using Lipofectamine 2000 reagent (Invitrogen) according to the manufacturer’s protocol.

### Isolation of total RNA and protein

Total RNA from GC cells in a 100 mm cell culture dish was prepared using Trizol reagent according to the manufacturer’s protocols (Invitrogen). Protein cell lysates were collected in RIPA buffer (Bio-rad). The supernatant was analyzed for protein content using the BCA method (Thermo Scientific).

### Real-time PCR and western analysis

Reactions were performed in triplicate for each sample using an ABI Power SYBR green PCR Master Mix (Applied Biosystems) with CHOP-specific primers [5′- ATGAGGACCTGCAAGAGGTCC-3′ (sense) and 5′- TCCTCCTCAGTCAGCCAAGC-3′ (antisense) and ATF4-specific primers (5′-AAGCCTAGGTCTCTTAGATG-3′ (sense) and 5′-TTCCAGGTCATCTATACCCA-3′ (antisense)] on a Roche LightCycler 96 System (Roche). RNA quantify was normalized to β-actin primers [5′-AAGGCCAAC CGCGAGAAGAT-3′ (sense) and 5′-TGATGACCTGGCCGTCAGG-3′ (antisense)], and gene expression was quantified according to the 2^−ΔCt^ method. To perform Western blot assay, GC cells were solubilized in the radioimmunoprecipitation assay (RIPA) lysis buffer (Bio-rad). The primary antibodies used were as follows: β-actin, Bcl-2, Beclin-1, ULK1, Atg5, GRP78, ATF4, CHOP, and caspase-12 and (Santa Cruz, 1:1000); LC3B and p62 (Sigma, 1:1000); CD63, IRE1α, p-IRE1α (S724) and G9a (Abcam, 1:1000); and cleaved caspase-3, caspase-9, -PARP, p62, AMPKα, p-AMPKα (Thr172), p-mTOR (Ser2448), p-p70S6K (Thr389), ULK1, p-ULK1 (Ser555), PERK, p-PERK (Thr980), eIF2α, p-eIF2α (Ser51), JNK, p-JNK (Thr183/Tyr185), STAT3, p-STAT3 (Tyr705), BNIP3, HIF-1α, H3K4me2, H3K9me2, H3K9me3, H3K27me2, H3K27me3, and H3 (CellSignaling, 1:1000). The blots were visualized by Western Chemiluminescent HRP Substrate (Millipore).

### Quantification of pEGFP–LC3 puncta

AGS and SNU-638 cells in a six-well plate were transfected with pEGFP–LC3 using Lipofectamin 2000 (Invitrogen), and then treated with SH003 (400 μg/mL) for 8 h. A pEGFP–LC3B-positive punctate pattern was observed by confocal microscopy (ZEISS LSM5 PASCAL).

### Immunoprecipitation

We extracted cell lysates from AGS and SNU-638 cells on a 100-mm cell culture plate in the immunoprecipitation (IP) buffer (Sigma). We incubated anti-Bcl-2 (Santa Cruz) and anti-Beclin-1 with lysate at 4 °C for 16 h. We used the protein A/G PLUS agarose (Santa Cruz) to pull down immunocomplexes.

### Chromatin immunoprecipitationassay

Chromatin immunoprecipitation (ChIP) assays were performed using an EZ ChIP Chromatin Immunoprecipitation kit (Millipore, Billerica, MA, USA) as described in the supplier’s protocol. Briefly, the cross-linked chromatin was sonicated after cell lysis and then incubated overnight at 4 °C with antibodies against ATF4 (SantaCruz), G9a (Abcam) and STAT3 (CellSignaling). The immunocomplex was precipitated with protein A–agarose (Millipore), and the beads were washed, sequentially treated with 10 µL of RNase A (37 °C for 30 min) and 75 µL of proteinase K (45 °C for 4 h), and incubated overnight at 65 °C to reverse cross-link the chromatin. The DNA was recovered by phenol–chloroform extraction and co-precipitation with glycogen and was then dissolved in 50 at 65 °C of Tris-EDTA (TE) buffer. DNA associated with the ER was amplified by PCR using 1 at 65 °C of precipitated DNA. PCR primers [5′-GAAGTGGCTATCGCCAGAGT-3′ (sense) and 5′- GCTGCTTGAAGGTCTTCTCC -3′ (antisense)] were designed to amplify the ATF4 and G9a binding site at the *LC3B* gene promoter and [5′-CTTTTCCCGCCTCTGGTTGCT-3′ (sense) and 5′-CTATCGCCCCTTCGTGCTCGT-3′ (antisense)] were designed to amplify the STAT3 binding site at the *G9a* gene promoter. Quantitative PCR conditions were 40 cycles at 94 °C for 40 s, 60 °C for 1 min, and 72 °C for 40 s.

### Exosome isolation

Exosomes were obtained from the supernatant of untreated and SH003 (0, 200, and 400 μg/mL)-treated AGS and SNU-638 cells according to the manufacturer’s protocols (Total Exosome Isolation Reagent (from cell culture media), Thermo Fisher Scientific).

### Statistical analysis

All results were confirmed in at least three independent experiments; Student’s *t*-tests were used for comparisons of means of quantitative data between groups and *p* < 0.05 was considered statistically significant.

## Supplementary information

Supplementary Information

Supplementary Figure 1.

Supplementary Figure 2.

Supplementary Figure 3.

Supplementary Figure 4.

Supplementary Figure 5.

Supplementary Figure 6.
