# Supplementary material for: SH003 activates autophagic cell death by activating ATF4 and inhibiting G9a under hypoxia in gastric cancer cells
Source: Cell Death Dis. 2020 Sep 2;11(8):717. doi: 10.1038/s41419-020-02924-w (PMC7468158; doi:10.1038/s41419-020-02924-w)
Supplement: Supplementary file 1 — Supplementary Information [file 41419_2020_2924_MOESM1_ESM.docx]

**Supplementary Materials: SH003 induces autophagic cell death by activating ATF4 and inhibiting G9a under hypoxia in gastric cancer cells.**

**Supplementary Figure Legends**

**Figure S1.**

**Autophagy inhibition induces cell survival flux in SH003-treated GC cells.** (A) AGS cells were pre-treated with BafA1 (10 nM) or CQ (20 μM) , and after SH003 treatment, cells were stained with Cyto-ID Green solution. After Cyto-ID stain, performed flow cytometry (FACs). **(**B) After AGS and SNU-638 cells were treated with BafA1 or CQ in SH003-treated AGS and SNU-638 cells, Western blotting analysis for LC3B protein levels were performed. (C) After AGS and SNU-638 cells were transfected by LC3B siRNA in AGS and SNU-638 cells, real-time RT-PCR for LC3B mRNA levels were investigated with SH003 (400 ug/mL, 24h) treatment; *, *p*<0.05. β-actin was used as a mRNA control.

**Figure S2.**

**Induction and inhibition of ER stress response regulate SH003-induced autophagic cell death in GC cells.** (A) Protein levels of p-IRE1α, IRE1α, p-JNK and JNK were determined using Western blotting analysis. (B) mRNA levels of ATF4 and CHOP were determined using real-time RT-PCR in Thapsigargin (3 μM, 24h) and SH003 (400 ug/mL, 24h)-treated GC cells; *, *p*<0.05.

**Figure S3.**

**Induction and inhibition of ER stress response regulate autophagic cell death in GC cells.** (A-C) Cell viability, LDH release, and western blot analysis of GRP78, PERK, p-eIF2α, ATF4, CHOP, and cleaved caspase-12 in the SNU-638 cells treated with SH003 (400 ug/mL, 24h) in the presence or absence of PERK inhibitor (10 μM, 24h); *, *p*<0.05. (D-F) Cell viability, LDH release and western blot analysis of CHOP, LC3B, cleaved caspase-3, and cleaved caspase-12 in the SNU-638 cells treated with SH003 (400 ug/mL, 24h) in the presence or absence of CHOP siRNA (30 nM, 24h); *, *p*<0.05. β-actin was used as a protein loading control.

**Figure S4.**

**Inhibition of ER stress response regulate autophagic cell death in GC cells.** (A and B) Cell viability and western blot analysis of p62, JNK, LC3B and cleaved caspase-3 in AGS cells treated with SH003 (400 ug/mL, 24h) in the presence or absence of SP600126 inhibitor (10 μM, 24h); *, *p*<0.05.

**Figure S5.**

**SH003 induces more autophagic cell death in hypoxia than normoxia.** (A) Expression of HIF-1α, BNIP3, ATG5, p62, and LC3B in SH003 (400 ug/mL, 24 h)-treated SNU-638 cells for indicated time on exposed conditions to normoxia or hypoxia. (B-D) SNU-638 cells were transfected with control or LC3B siRNA and then exposed to normoxia or hypoxia for 24 h in the presence of SH003 (400 ug/mL, 24 h). Cell viability, LDH assay, and western blotting were performed on this condition.

**Figure S6.**

**SH003 regulates STAT3-G9a axis in GC cells.** (A and B) The localization of the STAT3 on the *G9a* promoter. SH003 regulates STAT3 binding at the *G9a* promoter. SH003 (400 μg/mL, 24 h) treatment was performed in AGS and SNU-638 cells, and real-time ChIP assays of the *G9a* promoter region were performed with STAT3 antibody. (C) AGS cells were transfected with control or STAT3 siRNA in the presence or absence of SH003 (400 μg/mL, 24 h), and then, Western blotting analysis were performed; *, *p*<0.05.
