# Supplementary figures and images for: SH003 activates autophagic cell death by activating ATF4 and inhibiting G9a under hypoxia in gastric cancer cells
Source: Cell Death Dis. 2020 Sep 2;11(8):717. doi: 10.1038/s41419-020-02924-w (PMC7468158; doi:10.1038/s41419-020-02924-w)

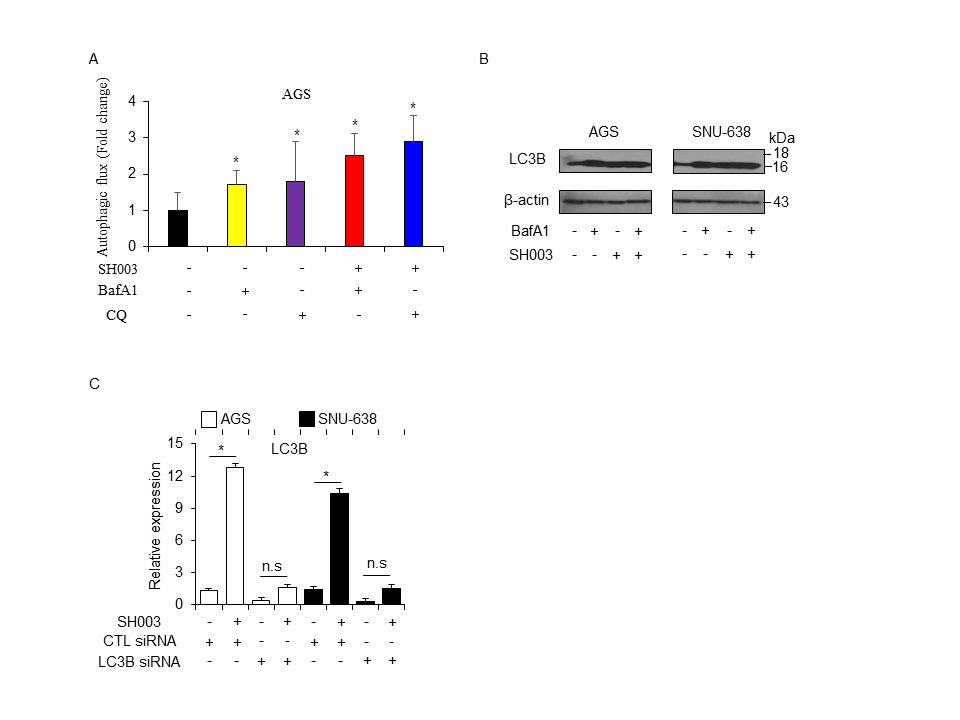

Supplement: Supplementary file 2 — Supplementary Figure 1. [file 41419_2020_2924_MOESM2_ESM.tif]

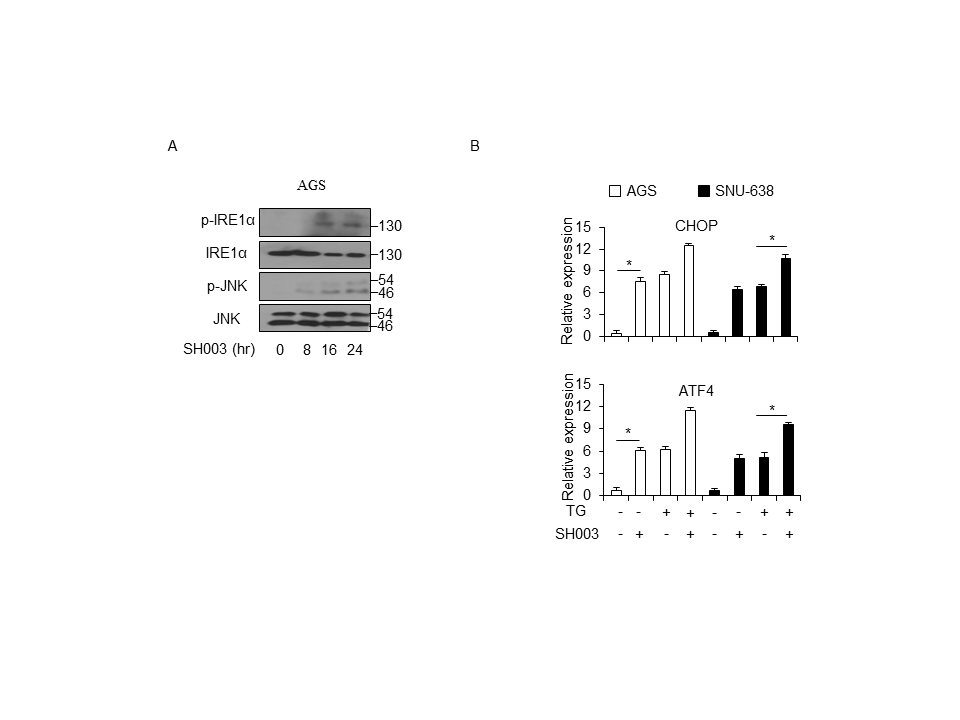

Supplement: Supplementary file 3 — Supplementary Figure 2. [file 41419_2020_2924_MOESM3_ESM.tif]

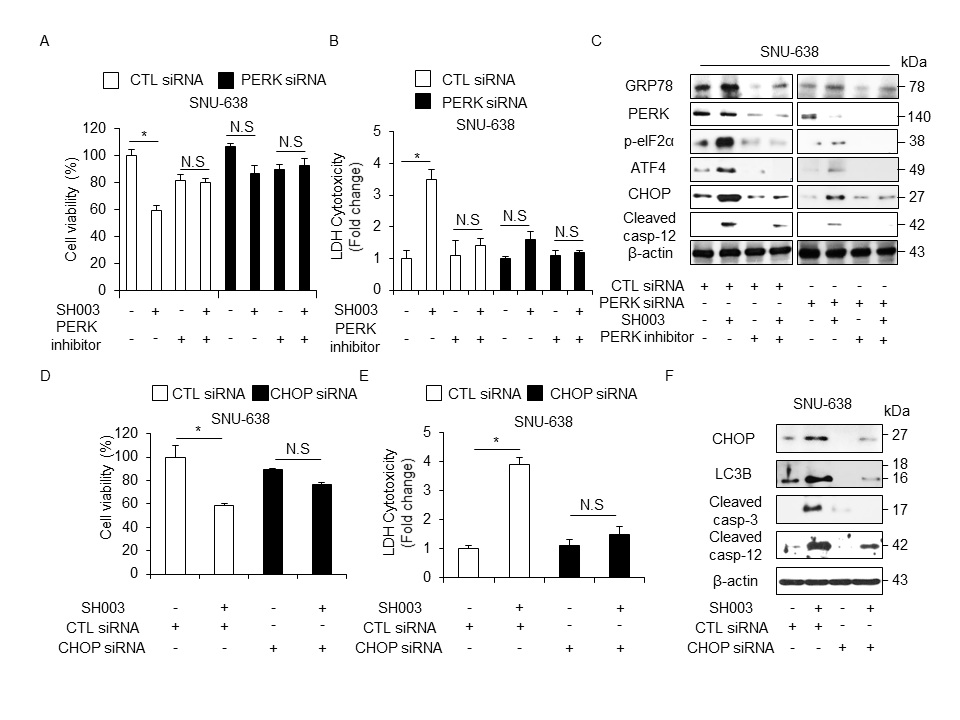

Supplement: Supplementary file 4 — Supplementary Figure 3. [file 41419_2020_2924_MOESM4_ESM.tif]

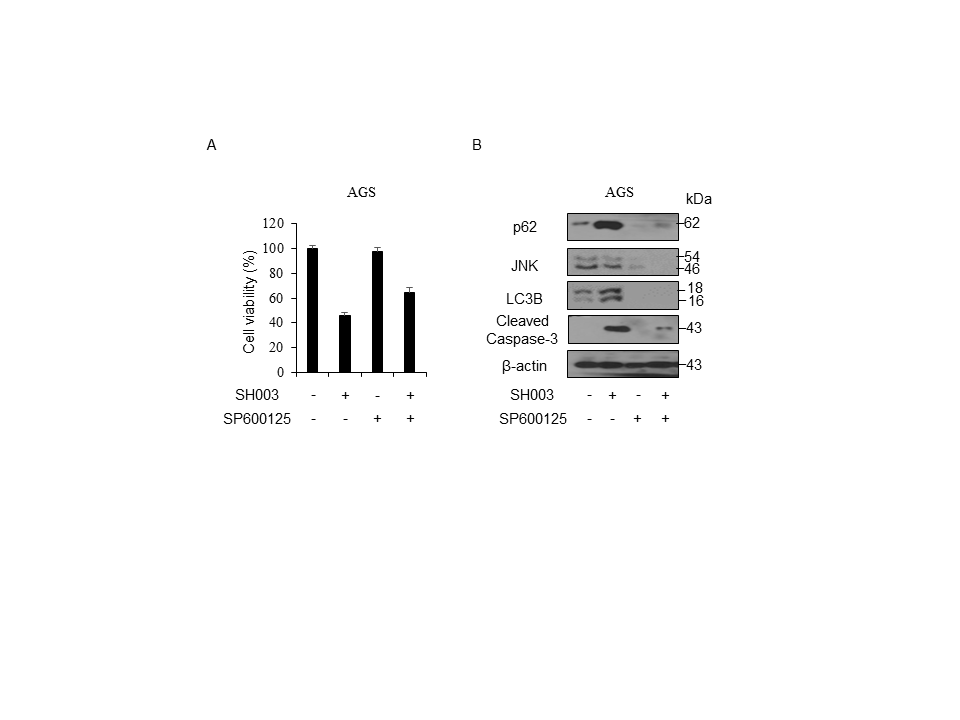

Supplement: Supplementary file 5 — Supplementary Figure 4. [file 41419_2020_2924_MOESM5_ESM.tif]

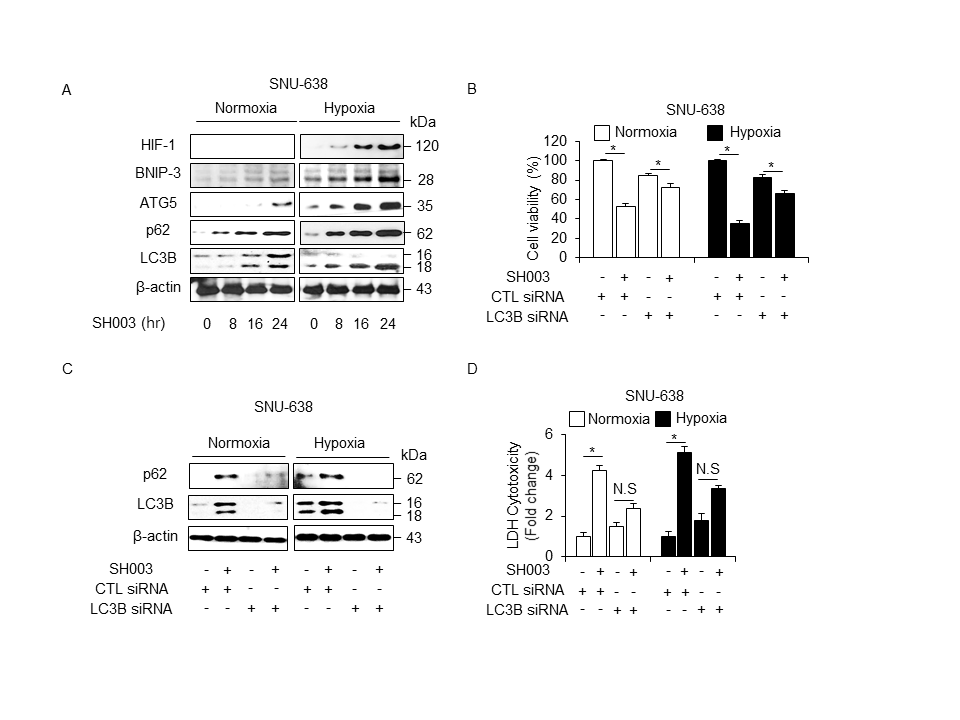

Supplement: Supplementary file 6 — Supplementary Figure 5. [file 41419_2020_2924_MOESM6_ESM.tif]

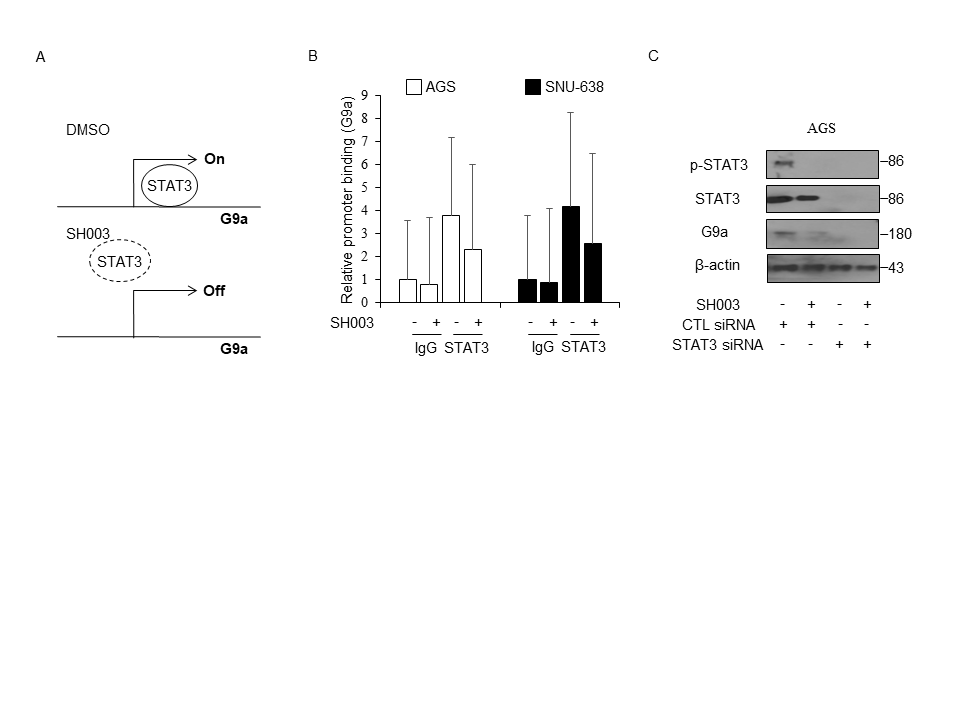

Supplement: Supplementary file 7 — Supplementary Figure 6. [file 41419_2020_2924_MOESM7_ESM.tif]
